# Synthesis of Main-Chain Chiral Quaternary Ammonium Polymers for Asymmetric Catalysis Using Quaternization Polymerization 

**DOI:** 10.3390/molecules17067569

**Published:** 2012-06-19

**Authors:** Naoki Haraguchi, Md. Masud Parvez, Shinichi Itsuno

**Affiliations:** Department of Environmental and Life Sciences, Graduate School of Engineering, Toyohashi University of Technology, 1-1 Hibarigaoka, Tenpaku-cho, Toyohashi, Aichi 441-8580, Japan

**Keywords:** chiral polymer, quaternization polymerization, organocatalysis, cinchona alkaloid, quaternary ammonium salt

## Abstract

Main-chain chiral quaternary ammonium polymers were successfully synthesized by the quaternization polymerization of cinchonidine dimer with dihalides. The polymerization occurred smoothly under optimized conditions to give novel type of main-chain chiral quaternary ammonium polymers. The catalytic activity of the polymeric chiral organocatalysts was investigated on the asymmetric benzylation of *N*-(diphenylmethylidene)glycine *tert-*butyl ester.

## 1. Introduction

Cinchona alkaloids are among the most attractive chiral molecules because they represent readily available and inexpensive natural chiral amino alcohols with pseudo-enantiomer forms. Cinchona alkaloids and their derivatives have been widely applied as chiral organocatalysts for some asymmetric reactions [1,2,3]. Among them, the quaternary ammonium salts of cinchona alkaloids [1,2,3,4] are widely used for asymmetric reactions such as alkylations [5,6], aldol reactions [7,8,9], nitroaldol reactions [7], Mannich reactions [10], nitro-Mannich reactions [11], Darzens reactions [12], Strecker reactions [13], α-hydroxylations [14], α-fluorinations [15], Michael reactions [16,17], epoxidations [18,19], aziridations [20,21], and reductions [22,23]. 

From the viewpoint of the practical use of cinchona alkaloid quaternary ammonium salts, some issues exist. For example, compared with transition metal based catalysts, large amounts (1–20 mol%) of organocatalyst molecule are generally required to complete the reaction, and only a few active cinchona alkaloid quaternary ammonium salts are reported. Moreover, due to the amphiphilicity of the quaternary ammonium salt, the catalyst separation might be difficult in some cases. One potential solution to the last problem is the immobilization on the catalyst on a polymer [1,4,24,25,26,27]. Several examples of polymer-immobilized chiral quaternary ammonium salts have been developed and successfully used as catalysts for asymmetric synthesis [28,29,30,31,32,33,34,35,36,37,38,39]. In these polymeric catalyst examples the catalyst was immobilized on the side-chain of the support polymer.

In addition of these polymer-immobilized chiral quaternary ammonium salts, we have recently developed main-chain chiral polymers with chiral organocatalysts [40,41,42,43,44,45]. Some useful polymerization techniques such as etherification polymerization [40], ion exchange polymerization [41,42,43,44], and neutralization polymerization [45] were used for the synthesis of main-chain chiral polymers with chiral organocatalysts. Interestingly, some main-chain chiral polymers showed higher enantioselectivity than the corresponding monomeric catalysts. 

In this article, some main-chain chiral quaternary ammonium polymers were synthesized by a quaternization polymerization of cinchonidine dimer with dihalides. The resulting main-chain chiral quaternary ammonium polymers were used as polymeric chiral organocatalysts for the asymmetric benzylation of *N*-(diphenylmethylidene)glycine *tert*-butyl ester.

## 2. Results and Discussion

### 2.1. Synthesis of Thiolated Cinchonidine Quaternary Ammonium Dimers ***5***

**Scheme 1:**
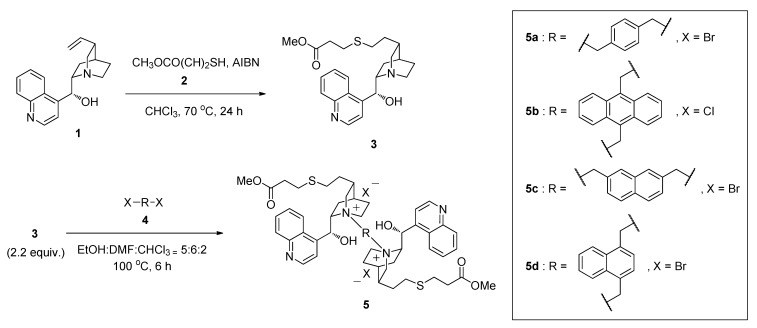
Synthesis of cinchonidine quaternary ammonium dimers **5**.

We have firstly prepared some thiolated cinchonidine quaternary ammonium salt dimers as model compounds. The synthetic scheme followed is shown in Scheme 1. The thiolated cinchonidine quaternary ammonium dimers **5** were prepared in two steps from cinchonidine (**1**), thiols **2** and dihalides **4**. The first thiol-ene click reaction of methyl-3-mercaptopropionate (**2**) and **1** was carried out in the presence of AIBN in CHCl_3_ at 70 °C for 24 h [46]. The reaction proceeded quantitatively and the thiolated cinchonidine **3** was obtained in 90% isolated yield. 

Dimerization of cinchona alkaloids was versatile because of the presence of some functional groups such as hydroxyl groups, the quinuclidine framework amine and even double bonds in cinchona alkaloids. Cinchona alkaloids are generally dimerized by etherification with dihalide to afford chiral diamine derivatives. The other dimerization method is a quaternization of cinchona alkaloids with dihalides to give chiral quaternary ammonium dimers. We employed the quaternization reaction for the synthesis of chiral quaternary ammonium dimers. The quaternization reaction was carried out at 100 °C using 2.2 equivalents of **3** to **4**. The reaction was complete within 6 h when the mixed solvent of ethanol/DMF/chloroform (5/6/2) was used. The isolated yields of **5a**–**d** were 92–96%. We found that cinchonidine could be easily modified with thiol via thiol-ene click reaction and the thiolated cinchonidine could be dimerized with dihalides by the quaternization reaction.

### 2.2. Synthesis of Cinchonidine Dimers ***7*** Using Thiol-Ene Click Reaction

**Scheme 2:**
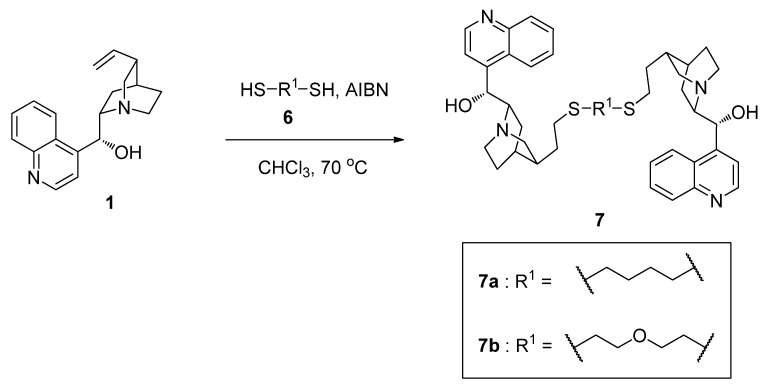
Synthesis of cinchonidine dimers **7**.

Another useful dimerization method we focused on here is the use of the double bond in cinchona alkaloids. Some useful coupling reactions such as thiol-ene click reaction [47,48,49,50] are available for the dimerization reaction. We tried to synthesize cinchonidine dimers **7a** and **7b** by the thiol-ene click reaction of **1** and dithiols **6**, as illustrated in Scheme 2.

The thiol-ene click reaction of two equivalents of cinchonidine with dithiol was carried out in the presence of AIBN in CHCl_3_ at 70 °C. Compared with the reaction of **1** and **2** in the synthesis of monothiolated cinchonidine **3**, the reaction with **6** was slow. In addition, we were faced with a problem in the purification of **7a** and **7b** by silica gel column chromatography. Large amounts of the product interacted with the stationary phase of column chromatography, which led to a decrease in the isolated yield of **7a** and **7b**.

### 2.3. Synthesis of Main-Chain Chiral Quaternary Ammonium Polymers ***8*** Using Quaternization Polymerization

**Scheme 3:**
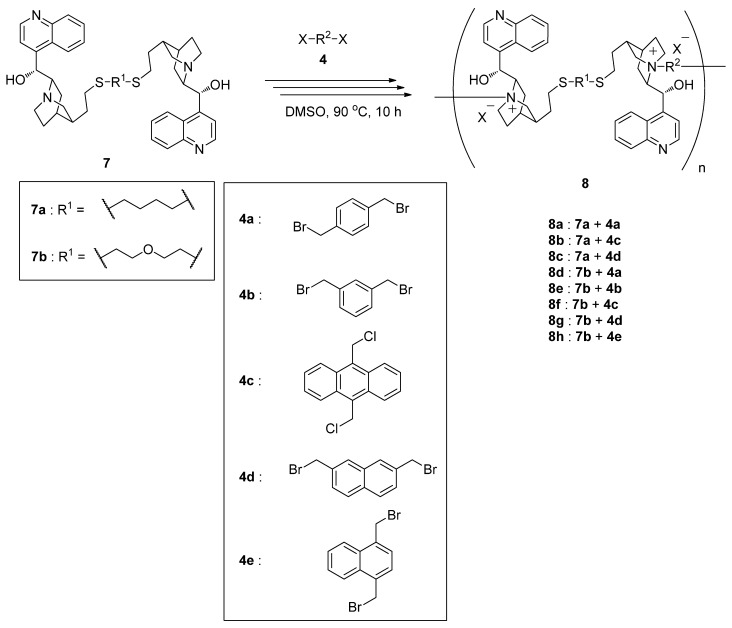
Synthesis of main-chain chiral quaternary ammonium polymers **8**.

We next tried to synthesize main-chain chiral quaternary ammonium polymers **8** using the thiolinked cinchonidine dimers **7**. The synthesis of **8** using quaternization polymerization is illustrated in Scheme 3. The procedure is quite simple. Compound **7** and equimolar dihalide **4** were reacted in DMSO at 90 °C. Neither initiator of the polymerization nor additives is necessary. In fact, the corresponding main-chain chiral quaternary ammonium polymers **8a**–**h** were obtained in high yield. In all cases polymeric products were precipitated into diethyl ether, which could be easily isolated by filtration.

*M*_n_ values of these polymers can be measured by SEC. The measured values in the elemental analysis of **8** were somewhat different from the calculated ones, possibly because of the hydrophilicity of the quaternary ammonium moiety and the low degree of polymerization. In the ^1^H-NMR spectra of the polymers, proton signals assigned to the quinuclidine framework and the dihalide disappeared completely. On the other hand, some signals assigned to the quaternary ammonium salt were alternatively observed. These results clearly indicated that successive intermolecular quaternization reaction occurred without side reaction participation to afford main-chain chiral quaternary ammonium polymers **8**. Due to the simplicity of the quaternization polymerization, various kinds of main-chain chiral quaternary ammonium polymers can be easily prepared by this method. In addition, this is the first successful report of the synthesis of main-chain polymers of cinchonidine quaternary ammonium salts using the double bond, in which the cinchonidine quaternary ammonium salts were incorporated uniformly into the main-chain of the polymer.

### 2.4. Asymmetric Benzylation of N-(Diphenylmethylidene)glycine tert*-*butyl Ester ***9*** Catalyzed by Main-Chain Chiral Quaternary Ammonium Polymers ***8***

Since these main-chain chiral quaternary ammonium polymers contain chiral quaternary ammonium structures in their repeating units, the polymers should have catalytic activity in some asymmetric transformations. In order to evaluate the catalytic activity of the new chiral ionic polymers, we chose the asymmetric benzylation of *N*-(diphenylmethylidene)glycine *tert*-butyl ester **9** as a typical asymmetric transformation using chiral quaternary ammonium salt. We designed and synthesized some main-chain chiral quaternary ammonium polymers based on various combinations of thiolinked cinchonidine dimers and dihalides to investigate the effect of the structure on the catalytic activity and the enantioselectivity.

At first, we have tested the asymmetric benzylation reaction with the chiral quaternary ammonium dimers **5a**–**d**. The results of the asymmetric reaction are summarized in Table 1. The substrate **9** was allowed to react in an organic solvent with benzyl bromide in the presence of the chiral quaternary ammonium dimer **5** and aqueous potassium hydroxide. In all cases, the asymmetric reaction took place smoothly in the two phase system. For example, chiral quaternary ammonium dimer **5a** catalyzed the benzylation to give the corresponding chiral (*S*)-phenylalanine derivative **10** in 97% yield with 84% *ee* (Table 1, entry 1). The structure of the spacer (*R*) between two chiral quaternary ammonium salts in the dimeric catalyst influences the enantioselectivity. When anthracenyl or naphthyl moieties were introduced as spacer, higher enantioselectivity (up to 90% *ee*) was observed (entries 2,3,4). In contrast, thiolated cinchonidine **3** and thiolinked cinchonidine dimers **7** did not show any catalytic activity at all. We thus confirmed that the chiral quaternary ammonium salt framework was essential to catalyze the asymmetric benzylation. 

**Table 1:** Asymmetric benzylation of glycine derivative using chiral quaternary ammonium dimer **5**.

Entry	Catalyst	R	Time (h)	Yield (%) ^a^	*ee* (%) ^b^
1	**5a**	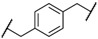	4	97	84
2	**5b**	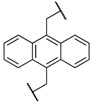	4	92	90
3	**5c**	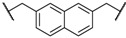	4	91	88
4	**5d**	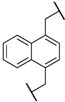	4	92	90 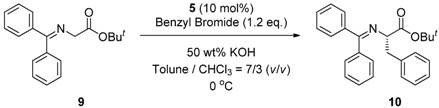

^a^ Determined by ^1^H-NMR; ^b^ Determined by HPLC (CHIRALCEL OD-H column).

Encouraged by these results in the asymmetric benzylation using chiral quaternary ammonium dimers **5**, main-chain chiral quaternary ammonium polymers **8a**–**h** were used as polymeric chiral organocatalysts in the asymmetric benzylation reaction under similar conditions in order to investigate their catalytic activity. The reaction conditions are as same as those using the dimeric catalyst. These results were summarized in Table 2. Compounds **8a**–**h** were not soluble, but were suspended in a mixed solvent of toluene/chloroform and aqueous potassium hydroxide. The benzylation reaction proceeded quantitatively without side reactions within 11 h. After the asymmetric benzylation reaction was complete, chiral product **10** was easily obtained by extracting the mixture with dichloromethane. Since these polymeric chiral catalysts were not soluble in the mixed solvent used in the reaction, these polymers could be easily separated from the reaction mixture. When **8a** was used as a polymeric chiral organocatalyst, the reaction occurred smoothly to afford **10** in 88% yield with 86% *ee* (entry 1). Unfortunately, the enantiomeric excess of **10** decreased to 79% when **8b,c** were used (entries 2 and 3). When **8d** with an ether linkage was used instead of **8a** with an alkyl one, both the yield and enantioselectivity increased slightly (*cf*. entry 1 *vs*. 4).

**Table 2:** Asymmetric benzylation of **9** using main-chain chiral quaternary ammonium polymer **8**.

Entry	Catalyst	R^1^	R^2^	Time (h)	Yield (%) ^a^	*ee* (%) ^b^
1	**8a**	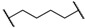	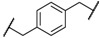	8	88	86
2	**8b**	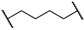	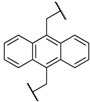	8	84	79
3	**8c**	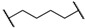	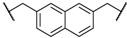	8	86	79
4	**8d**	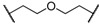	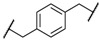	11	89	87
5	**8e**	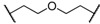	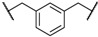	5	53	71
6	**8f**	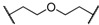	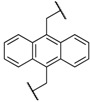	8	75	80
7	**8g**	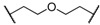	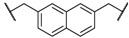	11	80	88
8	**8h**	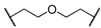	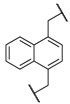	8	85	88 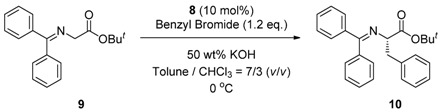

^a^ Determined by ^1^H-NMR; ^b^ Determined by HPLC (CHIRALCEL OD-H column).

Using the ether linkage as R^1^, the effect of R^2^ on the catalytic activity was also investigated. Enantioselectivity decreased significantly with the use of *m*-xylene-linked **8e** (entry 4 *vs*. 5). In contrast, naphthyl-linked **8g,h** showed higher enantioselectivity, which is comparable to the corresponding dimeric catalyst **5c** (entries 7 and 8). These results clearly indicated that these main-chain chiral quaternary ammonium polymers **8** showed good catalytic activity in the asymmetric benzylation of *N*-(diphenylmethylidene)glycine *tert*-butyl ester (**9**) and the modification of the structure of R^1^ and R^2^ in **8** is of significant importance for the design of main-chain chiral quaternary ammonium polymers as polymeric chiral catalysts. The catalytic activity of **8** was comparable to that of the other main-chain chiral quaternary ammonium polymers that were prepared by a successive etherification of cinchonidine quaternary ammonium salt dimers with dihalides [40].

## 3. Experimental

### 3.1. General

All solvents and reagents were purchased from Sigma-Aldrich, Wako Pure Chemical Industries, Ltd., or Tokyo Chemical Industry Co., Ltd. at the highest available purity and used as is, unless noted otherwise. Reactions were monitored by thin-layer chromatography (TLC) using Merck precoated silica gel plates (Merck 5554, 60F254). Column chromatography was performed on a silica gel column (Wakogel C-200, 100–200 mesh). Melting points were recorded using a Yanaco micro melting apparatus and are uncorrected. ^1^H- (300 MHz) and ^13^C-NMR (75 MHz) spectra were measured on a Varian Mercury 300 spectrometer. Peaks were referenced to the (CH_3_)_4_Si) (TMS) peak at δ = 0 (^1^H) or the solvent peak [*i.e*., the CDCl_3_ one at δ = 77. 1 (^13^C) or the DMSO ones at δ = 2.5 (^1^H)/39.5 (^13^C)]. The *J* values are reported in Hertz. IR spectra were recorded with a JEOL JIR-7000 FT-IR spectrometer and are reported in reciprocal centimeters (cm^−^^1^). Elemental analyses were performed at the Microanalytical Center of Kyoto University. Size exclusion chromatography (SEC) was performed with a Tosoh instrument with HLC 8020 UV (254 nm) or refractive index detection. DMF was used as a carrier solvent at a flow rate of 1.0 mL/min at 40 °C. Two polystyrene gel columns of bead size 10 μm were used. A calibration curve was made to determine number-average molecular weight (*M*_n_) and molecular weight distribution (*M*_w_/*M*_n_) values with polystyrene standards. HPLC analyses were performed with a JASCO HPLC system composed of a 3-line degasser DG-980, HPLC pump PV-980, column oven CO-965, equipped with a chiral column (CHIRALCEL OD-H or OD, Daicel) using hexane/2-propanol as eluent. A UV detector (JASCO UV-975 for the JASCO HPLC system) was used for the peak detection. Optical rotations were recorded with a JASCO DIP-149 digital polarimeter using a 10 cm thermostated microcell.

### 3.2. Preparation of Thiolated Chiral Quaternary Ammonium Salt Dimer ***5***

#### 3.2.1. Preparation of Thiolated Cinchonidine **3**

A mixture of cinchonidine (**1**, 3.53 g, 12.0 mmol), methyl-3-mercaptopropionate (**2**, 2.16 g, 18.0 mmol), and AIBN (0.039 g, 0.24 mmol) was stirred at 70 °C for 30 h. After the removal of the solvent under high vacuum, the residue was washed several times with hexane. The product **3** (4.48 g, 90% yield) was used for next reaction without further purification. m.p. 142 °C; [*α*]_D_^25^ = −52.50 (1.0, DMSO); ^1^H-NMR (CDCl_3_, TMS): δ = 1.38–1.55 (m, 4H), 1.63 (s, 1H), 1.74–1.80 (m, 3H), 2.31–2.38 (m, 3H), 2.43–2.56 (m, 2H), 2.67–2.73 (m, 2H), 2.98–3.06 (m, 2H), 3.40–3.51 (m, 1H), 5.63–5.73 (m, 1H), 7.34–7.40 (m, 1H), 7.55 (d, *J* = 4.8 Hz, 1H), 7.60–7.66 (m, 1H), 7.95 (d, *J* = 8.1 Hz, 1H), 8.05–8.08 (m, 1H), 8.79 (d, *J* = 4.8 Hz, 1H); ^13^C-NMR (CDCl_3_): δ = 20.97, 25.76, 27.10, 28.02, 30.19, 34.42, 34.60, 34.67, 43.28, 51.87, 58.22, 60.27, 71.52, 118.40, 123.05, 125.64, 126.71, 129.11, 130.09, 148.04, 150.02, 172.43; IR (KBr): ν = 1,585 (C=N), 1,220 (C-N), 1,130 (C-O), 750 (C-S); Elem. Anal. calc. for C_23_H_30_N_2_O_3_S: C 66.64, H 7.29, N 6.76; found C 66.50, H 7.27, N 6.62.

#### 3.2.2. Preparation of Thiolated Chiral Quaternary Ammonium Salt Dimer **5**

A mixture of **3** (0.456 g, 1.10 mmol) and **4** (0.50 mmol) in EtOH/DMF/CHCl_3_ = 5:6:2 mixed solvent (3 mL) was stirred at 100 °C for 6 h. After cooling the reaction mixture to room temperature, the mixture was dissolved in MeOH (2.5 mL) and poured into Et_2_O (20 mL). The mixture was stirred at room temperature for 1 h and filtered. The resulting solid was washed with Et_2_O several times to give the product **5**.

*Thiolated chiral quaternary ammonium salt dimer* **5a**: 96% yield. m.p. 189 °C; [*α*]_D_^25^ = −102.63 (1.0, DMSO); ^1^H-NMR (DMSO-d_6_): δ = 1.44 (br, 3H), 1.79 (br, 1H), 2.03 (br, 4H), 2.34–2.40 (m, 2H), 2.52–2.58 (m, 2H), 3.98 (d, *J* = 7.2 Hz, 2H), 4.32 (br, 2H), 5.02 (d, *J* = 11.4 Hz, 1H), 5.30 (d, *J* = 12.3 Hz, 1H), 6.65 (s, 1H), 7.04 (s, 1H), 7.88 (br, 3H), 7.96–8.00 (m, 2H), 8.21–8.24 (m, 1H), 8.45 (d, *J* = 8.4 Hz, 1H), 9.14 (d, *J* = 4.2 Hz, 1H); ^13^C-NMR (DMSO-d_6_): δ = 20.50, 23.39, 24.36, 25.73, 28.00, 32.19, 32.33, 33.79, 61.02, 61.63, 63.92, 67.15, 120.20, 124.09, 124.30, 126.46, 128.10, 129.40, 130.86, 133.73, 143.53, 147.75, 149.31, 171.55; IR (KBr): ν = 1,730 (-COOR), 1,633 (C=N), 1,218 (C-N), 1,040 (C-O), 759 (C-S); Elem. Anal. calc. for C_54_H_68_Br_2_N_4_O_6_S_2_: C 59.33, H 6.27, N 5.13; found C 58.49, H 6.34, N 5.14. 

*Thiolated chiral quaternary ammonium salt dimer* **5b**: 92% yield. m.p. 166 °C; [*α*]_D_^25^ = −215.81 (1.0, DMSO); ^1^H-NMR (DMSO-d_6_): δ = 1.19 (br, 3H), 1.43–1.51 (m, 3H), 1.86–1.96 (br, 2H), 2.18–2.25 (br, 2H), 2.40–2.44 (br, 2H), 4.48–4.55 (br, 2H), 5.93–5.98 (br, 1H), 6.36–6.55 (br, 1H), 7.11 (br, 1H), 7.72–8.04 (m, 5H), 8.14 (br, 1H), 8.25–8.33 (m, 1H), 8.79–8.84 (m, 1H), 8.97 (d, *J* = 7.5 Hz, 1H), 9.06–9.12 (m, 1H), 9.20 (d, *J* = 3.9 Hz, 1H); ^13^C-NMR (DMSO-d_6_): δ = 17.03, 21.22, 22.46, 24.04, 24.72, 25.82, 28.10, 31.96, 32.35, 32.78, 33.72, 33.83, 51.09, 54.33, 59.01, 62.02, 65.02, 65.60, 67.06, 119.02, 120.61, 123.89,124.65, 125.73, 126.29, 128.16, 131.14, 131.53, 132.52, 143.63, 147.45, 150.48, 171.53; IR (KBr): ν = 1,730 (-COOR), 1,625 (C=N), 1,215 (C-N), 1,038 (C-O), 779 (C-S); Elem. Anal. calc. C_62_H_72_Cl_2_N_4_O_6_S_2_: C 67.43, H 6.57, N 5.07; found C 65.95, H 7.03, N 4.98.

*Thiolated chiral quaternary ammonium salt dimer* **5c**: 93% yield. m.p. 192 °C; ^1^H-NMR (DMSO-d_6_): δ = 1.46 (br, 4H), 1.72 (br, 1H), 2.05 (br, 5H), 2.37–2.41 (m, 2H), 2.57 (d, *J* = 6.0 Hz, 2H), 4.01 (br, 1H), 4.38 (br, 1H), 5.12 (d, *J* = 12.9 Hz, 1H), 5.37 (d, *J* = 12.9 Hz, 1H), 6.63 (s, 1H), 6.79 (s, 1H), 7.74 (d, *J* = 6.0 Hz, 1H), 7.84 (d, *J* = 3.9 Hz, 2H), 7.94 (d, *J* = 7.8 Hz, 1H), 8.13 (d, *J* = 8.4 Hz, 1H), 8.22 (d, *J* = 7.8 Hz, 1H), 8.33 (d, *J* = 7.8 Hz, 2H), 9.01 (d, *J* = 3.9 Hz, 1H); ^13^C-NMR (DMSO-d_6_): δ = 20.52, 23.53, 24.54, 25.74, 28.02, 32.00, 32.47, 33.85, 50.58, 51.12, 61.19, 62.32, 63.79, 67.40, 119.97, 123.64, 124.17, 126.24, 127.05, 128.03, 129.18, 129.53, 131.48, 131.85, 134.14, 145.14, 147.33, 149.89, 171.62; IR (KBr): ν = 1,730 (-COOR), 1,617 (C=N), 1,235 (C-N), 1,022 (C-O), 777 (C-S); Elem. Anal. calc. C_58_H_70_Br_2_N_4_O_6_S_2_: C 60.94, H 6.17, N 4.90; found C 59.81, H 6.29, N 4.91.

*Thiolated chiral quaternary ammonium salt dimer* **5d**: 96% yield. m.p. 190 °C; ^1^H-NMR (DMSO-d_6_): δ = 1.41–1.46 (m, 4H), 1.74 (br, 1H), 1.97–2.03 (br, 4H), 2.18 (br, 1H), 2.32 (d, *J* = 6.3 Hz, 2H), 4.26 (br, 1H), 4.41 (br, 1H), 5.37 (d, *J* = 12.9 Hz, 1H), 5.81 (d, *J* = 12.0 Hz, 1H), 6.78 (s, 1H), 6.93 (d, *J* = 3.9 Hz, 1H), 7.79–7.90 (m, 4H), 8.13–8.16 (m, 2H), 8.44 (d, *J* = 8.4 Hz, 1H), 8.64 (br, 1H), 9.03 (d, *J* = 4.5 Hz, 1H); ^13^C-NMR (DMSO-d_6_): δ = 20.78, 23.16, 24.53, 25.84, 28.12, 32.39, 32.61, 33.84, 50.76, 51.14, 58.74, 61.49, 64.18, 67.43, 120.11, 123.90, 124.27, 125.13, 126.97, 127.25, 128.99, 129.56, 133.37, 133.64, 146.16, 146.69, 149.56, 171.60; IR (KBr): ν = 1,730 (-COOR), 1,632 (C=N), 1,235 (C-N), 1,062 (C-O), 758 (C-S); Elem. Anal. calc. C_58_H_70_ Br_2_N_4_O_6_S_2_: C 60.94, H 6.17, N 4.90; found C 59.77, H 6.18, N 4.96.

### 3.3. Preparation of Main-Chain Chiral Quaternary Ammonium Polymers ***8***

#### 3.3.1. Preparation of Cinchonidine Dimer **7**

A mixture of cinchonidine **1** (1.18 g, 4.00 mmol), dithiol (2.00 mmol), AIBN (6.4 mg, 0.04 mmol), and CHCl_3_ (6 mL) was stirred at 70 °C. During the reaction AIBN (0.04 mmol) was added three times successively. The residue after the removal of solvent under high vacuum was purified by column chromatography to give the product **7**.

*Cinchonidine dimer* **7a**: 40% yield. m.p. 116 °C; [*α*]_D_^25^ = −59.56 (1.0, DMSO); ^1^H-NMR (CDCl_3_, TMS): δ = 1.32–1.56 (m, 5H), 1.61–1.64 (m, 4H), 1.72–1.79 (m, 3H), 2.29–2.48 (m, 6H), 2.92–3.02 (m, 2H), 3.44–3.46 (m, 1H), 5.05 (s, 1H), 5.62 (s, 1H), 7.33–7.41 (m, 1H), 7.52–7.55 (m, 1H), 7.59–7.66 (m, 1H), 7.96–7.98 (m, 1H), 7.98–8.08 (m, 1H), 8.73–8.78 (m, 1H); ^13^C-NMR (CDCl_3_): δ = 20.54, 25.55, 27.84, 28.36, 29.87, 31.57, 34.57, 43.07, 49.81, 58.08, 60.08, 71.11, 118.27, 122.91, 125.46, 126.52, 128.94, 129.65, 147.65, 149.65, 150.40; IR (KBr): ν = 1,580 (C=N), 1,225 (C-N), 1,120 (C-O), 755 (C-S); Elem. Anal. calc. C_42_H_54_N_4_O_2_S_2_: C 70.95, H 7.65, N 7.88; found C 70.39, H 8.01, N 7.71. 

*Cinchonidine dimer* **7b**: 37% yield (for 70 h). m.p. 109 °C; [*α*]_D_^25^ = −52.57 (1.0, DMSO); ^1^H-NMR (CDCl_3_, TMS): δ = 1.37–1.49 (br, 4H), 1.58–1.76 (br, 5H), 2.30–2.42 (m, 4H), 2.54–2.58 (m, 2H), 2.91–3.02 (m, 2H), 3.46–3.52 (m, 4H), 5.35 (s, 1H), 5.65 (s, 1H), 7.35–7.40 (m, 1H), 7.54 (d, *J* = 4.2, 1H), 7.61–7.66 (m, 1H), 7.99–8.07 (m, 2H), 8.72 (d, *J* = 4.5, 1H); ^13^C-NMR (CDCl_3_): δ = 20.89, 25.64, 28.02, 30.63, 31.61, 34.61, 38.56, 43.23, 58.23, 60.24, 69.22, 70.76, 70.86, 71.40, 118.42, 123.04, 125.66, 126.74, 129.14, 129.98, 147.93, 149.90, 150.28; IR (KBr): ν = 1,590 (C=N), 1,235 (C-N), 1,100 (C-O), 760 (C-S); Elem. Anal. calc. C_42_H_54_N_4_O_3_S_2_: C 69.38, H 7.49, N 7.71; found C 69.02, H 7.70, N 7.61. 

#### 3.3.2. Preparation of Main-Chain Chiral Quaternary Ammonium Polymers **8**

A mixture of cinchonidine dimer **7** (0.500 mmol), dihalide **4** (0.500 mmol) in DMSO (2 mL) was stirred at 90 °C for 10 h. The residue after the removal of solvent under high vacuum was dissolved in MeOH (5 mL) and poured into Et_2_O (120 mL). After the filtration of the mixture, the solid was washed with hexane and ethyl acetate, and was dried under high vacuum to give the polymer **8**. 

*Main-chain chiral quaternary ammonium polymers* **8a**: 88% yield. [*α*]_D_^25^ = −117.1 (1.0, DMSO); *M*_n_(SEC) = 3.1 kg/mol; Elem. Anal. calc. C_50_H_62_Br_2_N_4_O_2_S_2_: C 61.59, H 6.41, N 5.75; found C 61.56, H 6.42, N 5.75.

*Main-chain chiral quaternary ammonium polymers* **8b**: 84% yield. *M*_n_(SEC) = 2.8 kg/mol; Elem. Anal. calc. C_70_H_64_Cl_2_N_4_O_2_S_2_: C 70.64, H 6.75, N 5.68; found C 69.93, H 6.75, N 5.68.

*Main-chain chiral quaternary ammonium polymers* **8c**: 93% yield. [*α*]_D_^25^ = −146.22 (1.0, DMSO); *M*_n_(SEC) = 3.6 kg/mol; Elem. Anal. calc. C_54_H_64_Br_2_N_4_O_2_S_2_: C 63.27, H 6.29, N 5.47; found C 62.47, H 6.62, N 5.46.

*Main-chain chiral quaternary ammonium polymers* **8d**: 96% yield. [*α*]_D_^25^ = −120.1 (1.0, DMSO); *M*_n_(SEC) = 10.8 kg/mol; Elem. Anal. calc. C_50_H_62_Br_2_N_4_O_3_S_2_: C 60.60, H 6.31, N 5.65; found C 60.78, H 6.23, N 5.62.

*Main-chain chiral quaternary ammonium polymers* **8e**: 96% yield. [*α*]_D_^25^ = −131.24 (1.0, DMSO); *M*_n_(SEC) = 6.8 kg/mol; Elem. Anal. calc. C_50_H_62_Br_2_N_4_O_3_S_2_: C 60.60, H 6.31, N 5.65; found C 61.10, H 6.21, N 5.61.

*Main-chain chiral quaternary ammonium polymers* **8f**: 67% yield. *M*_n_(SEC) = 2.8 kg/mol; Elem. Anal. calc. C_58_H_66_Cl_2_N_4_O_3_S_2_: C 69.51, H 6.64, N 5.59; found C 70.54, H 6.54, N 5.53.

*Main-chain chiral quaternary ammonium polymers* **8g**: 90% yield. [*α*]_D_^25^ = −135.48 (1.0, DMSO); *M*_n_(SEC) = 3.7 kg/mol; Elem. Anal. calc. C_54_H_64_Br_2_N_4_O_3_S_2_: C 62.30, H 6.20, N 5.38; found C 62.43, H 6.22, N 5.48.

*Main-chain chiral quaternary ammonium polymers* **8h**: 96% yield. [*α*]_D_^25^ = −139.7 (1.0, DMSO); *M*_n_(SEC) = 4.3 kg/mol; Elem. Anal. calc. C_54_H_64_Br_2_N_4_O_3_S_2_: C 62.30, H 6.20, N 5.38; found C 62.23, H 6.22, N 5.39.

### 3.4. General Procedure for Asymmetric Benzylation of N-(Diphenylmethylidene)glycine tert*-*butyl Ester ***9*** Catalyzed by Main-Chain Chiral Quaternary Ammonium Polymers ***8***

Main-chain chiral quaternary ammonium polymer **8** (0.018 mmol) and *N*-(diphenyl-methylidene)glycine *tert-*butyl ester (**9**, 0.053 g, 0.180 mmol) were added into a mixed solvent of toluene (7 mL) and chloroform (3 mL). 50 wt% aqueous KOH solution (2.5 mL) was added to the above mixture. Benzyl bromide (0.037 g, 0.216 mmol) was then added dropwise at 0 °C to the mixture. The reaction mixture was stirred vigorously at 0 °C. The reaction was monitored by TLC. After completion of the reaction, 10 mL of saturated sodium chloride solution was added, and the mixture was subsequently filtered to recover **8**, which was washed with water and dichloromethane several times. The organic phase was separated, and the aqueous phase was extracted with dichloromethane. The organic extracts were washed with brine and dried over MgSO_4_. Evaporation of solvents and purification of the residual oil by column chromatography on silica gel (diethyl ether:hexane = 1:10 as eluent) gave (*S*)-phenylalanine derivative **10**. The enantiomeric excess was determined by HPLC analysis (Daicel Chiralcel OD-H, hexane/2-propanol = 100:1, flow rate = 0.3 mL/min, retention time: *R* enantiomer = 27.6 min, *S* enantiomer = 47.9 min). The absolute configuration was determined by comparison of the HPLC retention time with the authentic sample independently synthesized by the reported procedure [6].

## 4. Conclusions

We have designed novel main-chain chiral quaternary ammonium salt polymers using thiol-ene click reaction followed by quaternization polymerization. The resulting main-chain chiral quaternary ammonium salt polymers have successfully been applied as polymeric chiral organocatalysts for the asymmetric benzylation of glycine derivatives. Thiolinked cinchonidine dimer could be synthesized by the thiol-ene click reaction of cinchonidine with dithiol. The quaternization polymerization with dihalide proceeded smoothly without side reactions to afford main-chain chiral quaternary ammonium salt polymers. We found that these main-chain chiral quaternary ammonium polymers showed good catalytic activity in the asymmetric benzylation of *N*-(diphenylmethylidene)glycine *tert*-butyl ester. Furthermore, the modification of the structure of R^1^ and R^2^ in **8** was significantly important for the design of main-chain chiral quaternary ammonium polymers as polymeric chiral catalysts. The effect of hydroxyl group functionalization and the reusability of the main-chain chiral quaternary ammonium salt polymers are now under investigation.
